# The validity of a free breathing motion corrected phase sensitive inversion recovery sequence in the detection of delayed myocardial enhancement in non-ischemic heart disease

**DOI:** 10.1186/1532-429X-16-S1-P305

**Published:** 2014-01-16

**Authors:** Oisin Flanagan, Bruce S Spottiswoode, Maria Carr, Jeremy D Collins, Xiaoming Bi, Marcos Botelho, Jad Bou Ayache, Robert R Edelman, James C Carr

**Affiliations:** 1 Radiology, Northwestern University Feinberg School of Medicine, Chicago, Illinois, USA; 2 Cardiovascular Imaging, Northwestern Memorial Hospital, Chicago, Illinois, USA; 3 Siemens Healthcare USA, Los Angeles, California, USA; 4 Siemens Healthcare, Chicago, Illinois, USA; 5 Radiology, Northshore University Healthsystem, Evanston, Illinois, USA

## Background

Breath-hold (BH) segmented TURBO FLASH (TFL) is currently used as the gold standard technique to evaluate delayed enhancement (DE) of the myocardium, typically with a phase sensitive inversion recovery (PSIR) approach (1). However, many patients are unable to perform adequate breath-holding resulting in poor image quality and limited diagnostic yield. Free breathing (FB) single shot steady state free precession (SSFP) is used as an alternative approach however respiratory motion artifact with resultant blurring may affect visualization of smaller myocardial scars. FB motion corrected (MOCO) single shot SSFP with averaging (2) has been shown to be equal or superior in detecting myocardial infarction (3), particularly in vulnerable patients. For FB MOCO sequences to replace current BH techniques in the detection of DE, they must be sensitive to the detection of both ischemic and non-ischemic patterns of delayed enhancement.

## Methods

16 consecutive patients, with suspected cardiomyopathy, who underwent cardiac MRI on a 1.5T system (MAGNETOM Aera, Siemens, Erlangen, AG) were imaged with FB PSIR SSFP, BH PSIR TFL and FB PSIR MOCO SSFP. Images were graded by an experienced cardiovascular physician for image quality (scale of 1 to 5), the presence of DE, localisation (sub endocardium, mid myocardium and sup epicardium), number of segments showing DE (0-17) and diagnostic confidence (scale of 1 to 3).

## Results

Image qualities for FB MOCO SSFP, FB single shot SSFP and BH TFL were 4.56, 4.31 and 3.78 respectively (Table 1). 6 of 18 patients (33.3%) demonstrated non-ischemic DE on all 3 sequences. In this subgroup, diagnostic confidences for FB MOCO, FB SSFP and BH TFL were 2.83, 2.67 and 2.33 respectively (Table 1). The total numbers of involved segments for the 3 techniques were 39, 38 and 36 respectively. Of the 39 DE segments identified on FB MOCO, 2 were not identified on FB SSFP and 4 were not identified on BH TFL. 1 DE segment was identified on each of FB SSFP and BH TFL (with low diagnostic confidence) but neither other sequence. In these 2 cases, additional segments showed DE which was detected on FB MOCO and diagnosis was unchanged. In every segment with DE identified on all 3 techniques, the volume of involved myocardium detected was equal or greater on FB MOCO than FB SSFP or BH TFL.

**Table 1:** Results of image quality and diagnostic confidence in all patients and in the subgroup of patients with non-ischemic delayed enhancement.

	Average Image quality **1=very poor and not analyzable, 2=poor, 3=acceptable, 4=good**, 5=very good	Average Diagnostic confidence; **1=low confidence**, **2=some confidence**, 3=high confidence
Total (n = 16)		

FB MOCO	4.56	N/A

FB SSFP	4.31	N/A

BH TFL	3.78	N/A

Non-ischemic DE (n = 6)		

FB MOCO	4.50	2.83

FB SSFP	4.00	2.67

BH TFL	3.50	2.33

## Conclusions

All patients with DE on either FB SSFP or BH TFL were identified on FB MOCO. More DE segments were identified on FB MOCO than either FB SSFP or BH TFL. FB MOCO provided equal or superior image quality and diagnostic confidence. While some variation with BH TRUFI and BH TFL was identified, image quality and diagnostic confidence was higher for FB MOCO than FB SSFP and BH TFL, which both showed low diagnostic confidence in these cases. While further study with larger numbers is required to validate these findings, initial experience suggests that FB MOCO is equal or superior to FB SSFP and BH TFL in detecting non-ischemic DE and could replace them in clinical practice.

## Funding

Research support from Siemens Healthcare.

**Figure 1:**
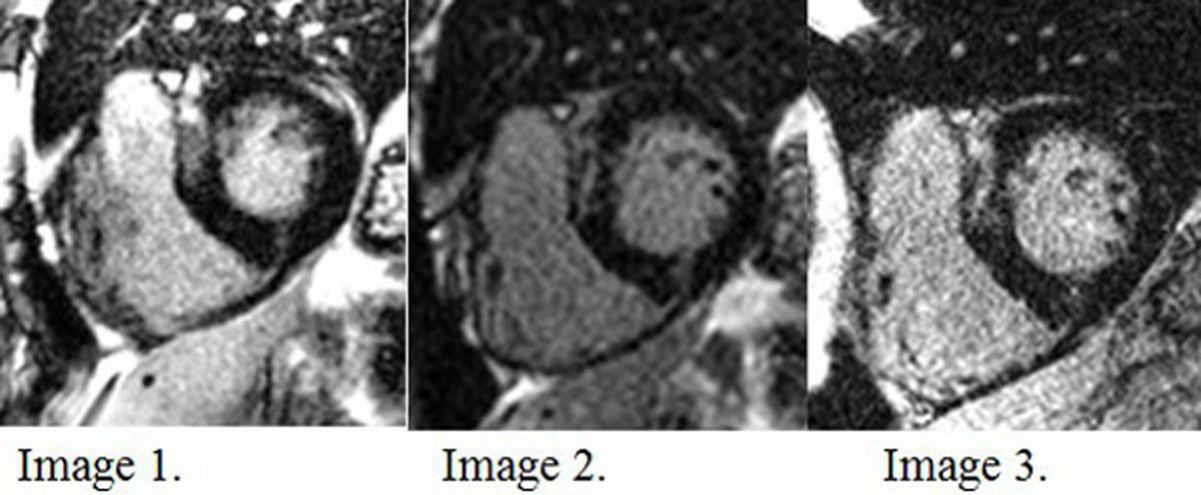
**FB MOCO PSIR**. Delayed enhancement noted in the basal anteroseptal and inferoseptal segments. Image quality 5 (very good) and diagnostic confidence 3 (high). Image 2. FB SSFP PSIR at same level. Image quality 4 (good) and diagnostic quality high (3). Image 3. BH TFL PSIR at same level. While the image quality is lower (4 (good) for this image but 3 overall for the sequence), the diagnostic confidence was 3 (high) in this case.

